# Intramolecular Pnictogen Bonds as Key Determinants for NMR Quantum Computation Parameters

**DOI:** 10.1021/acsomega.5c05640

**Published:** 2025-09-16

**Authors:** Gustavo A. Andolpho, Teodorico C. Ramalho

**Affiliations:** 1 Chemistry Department, Institute of Natural Sciences, 67739 Lavras Federal University, Lavras, MG 37200-900, Brazil; 2 Center for Basic and Applied Research, University Hradec Kralove, Hradec Kralove 500 03, Czech Republic

## Abstract

In this work, four molecules, two naphthalene derivatives and two acenaphthene derivatives, were studied via DFT for their ability to act as a quantum bit (qubit) for transferring information for NMR quantum computational information (QIP). NMR calculations indicate that all four molecules are suitable as qubits. Additionally, AIM, NBO, and EDA analyses provided insights into the presence and nature of intramolecular interactions between key atoms relevant to NMR-QIP. The results suggest that these P—P or P—Se interactions correspond to pnictogen bonds (PnB) in three compounds and to the chalcogen bond in the other compound, with most of their interaction energy originating from orbital interactions. To investigate the role of PnB in NMR parameters, the P—P interaction was modified to either increase or decrease its interaction energy. AIM and EDA analyses, combined with NMR calculations, reveal that as the interaction strengthens the NMR parameters become more suitable for NMR-QIP. Additionally, the results confirm that orbital interactions remain the primary contributor to the interaction energy. In summary, the findings of this study highlight the relationship between intramolecular pnictogen interactions and NMR parameters in four compounds, with potential applications in quantum information processing.

## Graphical abstract

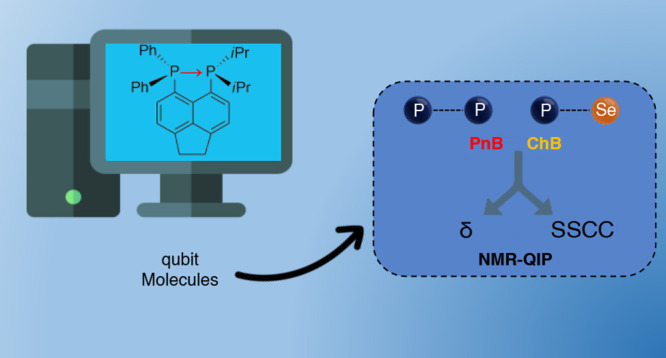

## Introduction

In recent years, the demand for quantum computers has been steadily rising as they have the potential to solve complex problems that have challenged society for decades. At the same time, artificial intelligence is becoming an integral part of our daily lives, further driving the need for advanced computing power. This ongoing technological evolution is shaping the future in ways we are only beginning to understand.^1^ To meet this demand, many studies have emerged on quantum information processing (QIP).^1–10^ One of the main techniques for QIP is nuclear magnetic resonance (NMR),^6–8,10^ which uses quantum bits (qubits) as the basis for transferring information.^7^ To do this, qubits need to have spin 1/2, so nuclei such as ^1^H, ^13^C, ^19^F, and ^31^P are some of the most common nuclei for this application.^4,5^ Other nuclei that also have this spin can be used for this purpose, such as ^113^Cd, ^199^Hg, or ^77^Se.^4,5^

One of the main challenges of quantum information processing is its scalability. As a result, research on building large-scale quantum processors using NMR has become increasingly intense. Scientists are continuously exploring more efficient ways to control quantum states while also striving to accommodate a greater number of qubits within the system.^4^ A good candidate for a qubit must be capable of implementing quantum gates, as well as being able to be put together in an organized and scalable way.^3,6,7,11^

To be applicable for this use, a molecule must fulfill the so-called DiVicenzo criteria,^5,12^ having a large difference between the chemical shifts of the nuclei involved and a high value of spin–spin coupling between these nuclei.^3–5^ Some studies have reported molecules that meet these criteria and can therefore be applied as qubits.^1,3–5^ Among these studies, it was possible to observe the use of molecules with naphthalene in their structure,^3^ as well as the use of P and Se atoms as the nuclei of interest for NMR.^3,5^

Therefore, the NMR parameters of these atoms of interest are of great importance for a molecule to be applicable as a qubit. It is also well known that these parameters are sensitive to intermolecular or intramolecular interactions that can occur in the molecule.^6,7,10,13–15^ Given that some of the good qubit molecules contain the P nucleus as the atom of interest for the NMR values, it is crucial to relate the NMR parameters to the interactions that occur with this atom, in particular the so-called pnictogen bond (PnB).^16,17^

Recently, this interaction has attracted a lot of attention in the literature due to its importance in various types of systems.^17–21^ Beyond pnictogen bonds, other types of directional interactions involving elements from groups 12 to 17 have also been widely explored. These include spodium, halogen, chalcogen, and tetrel bonds, which, despite involving different atomic centers, share key features with pnictogen bonds, such as their highly directional nature and dependence on electrostatic regions like σ-holes or π-hole.^19,22–26^ These regions act as localized sites of positive electrostatic potential that can engage in attractive interactions with electron-rich atoms or lone pairs. The comparative analysis of these interactions, both experimentally and through DFT-based methods, has contributed to a broader understanding of how orbital and electrostatic effects combine to determine interaction strength and spectroscopic behavior.^13–15,22,23,27^ This broader framework reinforces the relevance of studying PnB not only as an isolated phenomenon but also as part of a general class of interactions that can impact molecular design and function.

In addition, new findings have emerged that point to doubt regarding the explanation of the nature of the PnB,^16,17,19,20^ where the σ-hole, i.e., the electrostatic factor, is used to justify the interaction between the atoms involved in the interaction.^24^ This doubt arises from studies that show that, not only in PnB but in other so-called noncovalent interactions, a large part of the interaction energy comes from the interaction between the Lewis orbitals of the acid and the base.^17,20^ Therefore, the strength and nature of the interactions that take place in molecules are important factors in understanding the behavior of these molecules in NMR and, consequently, their use for qubits.

The compounds investigated in this study were selected based on the presence of well-defined spin-active nuclei and well-characterized NMR behavior, as well as the occurrence of intramolecular interactions involving P and Se atoms, which can be analyzed in terms of pnictogen and chalcogen bonding. In this line, two compounds with substituted naphthalene^28,29^ and two with substituted acenaphthene^30,31^ that are suitable for QIP and present the PnB are shown in Figure 1. These four compounds were selected for this study to investigate, via DFT,^32^ the influence of the pnictogen bond on the spin–spin coupling constants (SSCC) and the chemical shift (δ) values, which are essential parameters for the QIP.^1,8,10^

**Figure 1:**
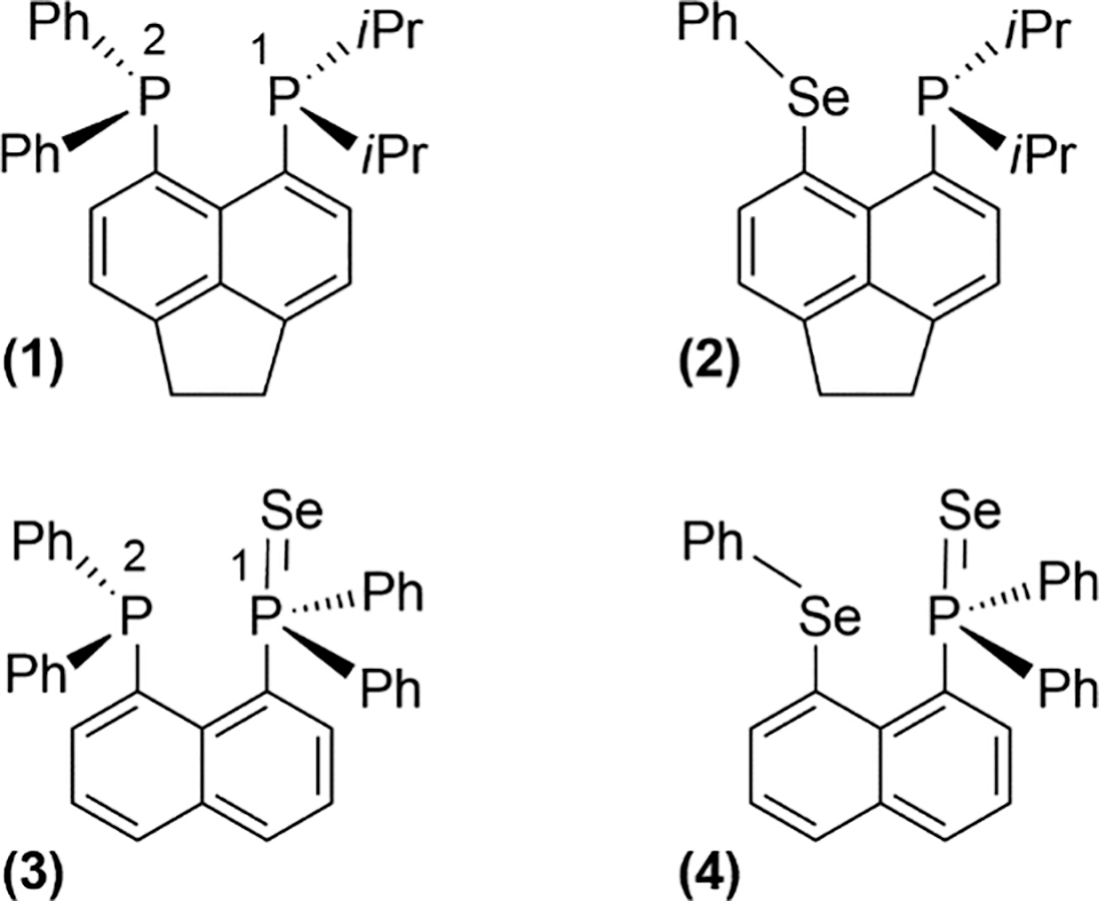
(**1**) Acenaphthene­(PPh_2_)­(P*i*Pr_2_); (**2**) acenaphthene­(SePh)­(P*i*Pr_2_); (**3**) naphthalene­(PPh_2_Se)­(PPh_2_); and (**4**) naphthalene­(PPh_2_Se)­(SePh).

## Methods

The crystallographic structures of compounds **1**–**4** were obtained from the Cambridge Crystallographic Data Base (CCDC codes = 1009481, 2278201, 145354, and 766926, respectively). These structures were optimized using density functional theory (DFT) with the PBE0 functional^33^ including relativistic effects (ZORA), ZORA-TZVP basis set,^34^ and D4 dispersion corrections. Optimized geometries were confirmed as a minimum by frequency analysis. This protocol was reported as an efficient methodology for the geometry optimization of similar compounds.^5^

NMR parameters, namely, chemical shifts (δ) and spin–spin coupling constants (SSCC or *J*), were calculated using B3LYP|aug-cc-pVTZ-J and PBE0-ZORA|pcJ-2 levels of theory, respectively. These protocols showed a successful performance for these parameters in these types of compounds.^3,35^ To obtain the δ, PH_3_ and SeC_2_H_6_ were used as reference,^5^ for P and Se, with chloroform as solvent using the CPCM solvation model.

Intramolecular interactions were characterized through atoms-in-molecules (AIM) analysis, performed using Multiwfn 3.7 software.^36^ The wfn-files were obtained through DFT calculations under the B3LYP-ZORA|ZORA-TZVP theory level.^34,37^ In the sequence, the NBO 6.0 program^38^ in the Gaussian09 package, using the B3LYP functional and def2-TZVP basis set, was used to perform the natural bond orbital (NBO) analysis. Finally, the AMS2022 package^39^ was used to perform the energy decomposition analysis (EDA) at the B3LYP|TZ2P theory level. These methods were selected due to their suitability for describing weak directional interactions such as PnB and ChB, allowing the assessment of their electronic nature and energetic contributions.^16–18,20^ To investigate the influence of these interactions on NMR parameters, internuclear distances were systematically varied at 0.1 Å intervals,^20^ with single-point calculations performed at each step. Unless otherwise specified, all DFT calculations were performed using the ORCA 5.0.4 package.^40^

## Results and Discussion

### Validation of NMR Calculations

The first step is to validate our methodology for obtaining the NMR parameters. Table 1 presents *J*_PP_, δ­(P1), and δ­(P2) values, for compounds **1** and **3** and *J*_PSe_, δ­(Se), and δ­(P) values for compounds **2** and **4**, calculated along with experimental values for these parameters, used as reference.

**Table 1:** Experimental and Theoretical NMR Parameters, *J*_PP_, δ­(P1), and δ­(P2), for compounds **1** and **3** and *J*_PSe_, δ­(Se), and δ­(P) for Compounds **2** and **4**

	**spin–spin coupling constant (Hz)**		**chemical shifts (ppm)**					
**compound**	**experimental** ^a^	theoretical (Δ)		**experimental** ^a^	theoretical (Δ)		**experimental** ^a^	theoretical (Δ)
**1**	160	163.99 (3.99)	P(1)	–11.3	2.61 (13.91)	P(2)	–12.8	–23.71 (10.91)
**2**	452.2	408.567 (43.63)	Se	425.3	540.00 (114.70)	P	–6.0	5.05 (11.05)
**3**	53	48.318 (4.68)	P(1)	42.8	72.99 (30.19)	P(2)	–8.7	1.57 (10.27)
**4**	715	766.288 (51.29)	Se	451.4	449.62 (1.78)	P	40.5	90.03 (49.53)

a Experimental values from references 26, 25, 23, and 24, for compounds **1–4**, respectively.

These values point out that our theoretical approach is suitable for obtaining the NMR parameters for compounds **1**–**4**. For the δ values, the error is, in general, smaller for phosphorus, especially when the scale for this parameter is considered.^41–46^ The error percentage related to the scale ranges from 1.8% for the P2 atom in compound **3** to 8.6% for the P atom in compound **4**. For the selenium atom, the error related to the scale is smaller than 0.5% for compound **4**, and around 4.4% for compound **2.**

The SSCC values present a similar behavior with the calculated *J*_PP_ with smaller errors than *J*_PSe_. Reported values for similar compounds point out that this error ranges from around 28 to 67 Hz for *J*_PSe_ ^4,47^ and 9 to 71 Hz for *J*_PP_.^3,48^ So, errors of around 4 Hz for compounds **1** and **3** *J*_PP_ and around 43 Hz for compound **2** and 51 Hz for compound **4** *J*_PSe_ are in agreement with the reported values and, therefore, show the effectiveness of the theoretical methodology chosen to obtain the SSCC values.^3,4,47,48^

### The Existence and Nature of Intramolecular Interactions and Their Relationship with NMR Quantum Computation Parameters

Intra­(inter)­molecular interactions are the name given to interactions similar to the hydrogen bond (HB), in which a Lewis acid interacts with a Lewis base, forming the interaction, which is classified according to the family of the atom in the periodic table.^14,24,49^ These include halogen (XB), chalcogen (ChB), pnictogen (PnB), and tetrel (TB) bonds.^14,19,49,50^ Understanding the nature of these interactions has attracted a lot of interest from the academic community in recent years,^14,16–20,24,49,50^ as controversies about the energy governing these interactions have arisen in recent years.^16–20,49^ Recent studies point out that the interaction energy between the σ* orbital of the covalent bond from the Lewis acid and the HOMO of the Lewis base is the main factor in the interaction energy between these fragments, i.e., a covalent component.^16,17^

Studying the nature of intramolecular interactions is a crucial factor when considering molecules for qubits, as the literature suggests that these interactions significantly influence the NMR parameters of the atoms involved.^14,50^ The relationship between the strength of the interaction and the NMR parameters is well-defined when related to Lewis bases; however, this correlation, although it exists, does not play a quantitative role in Lewis’s acid analysis.^14,50^

Recently, two studies have made a direct correlation between the NMR parameters and the interaction data provided by AIM calculations, for systems where there is HB, XB, ChB, PnB, or TB, occurring between Me_2_CO and 20 different Lewis acids in one study^14^ and another study with (CH_3_)_3_PSe interacting with the same 20 Lewis acids,^50^ pointing to a high correlation between the interaction energy and variation of shield tensor (Δσ) parameters for C and O in the first study, and the second study for the Δ*J*_PSe_ value and some correlation with the Δδ­(P) and Δδ­(Se) values.

Also, the pnictogen bond has been investigated in several chemical phenomena showing its importance; however, there is a lack of NMR studies that consider how this type of interaction affects the NMR parameters. With this in mind, it is important to understand the molecular interaction, likely a pnictogen bond (PnB) formation, that occurs between the phosphorus atoms in compounds **1** and **3**, where one P could act as the Lewis basis and the other as the Lewis acid, or for compounds **2** and **4**, between P and Se, where the Se atom could act as the donor atom and the P as the receiving atom.

Employing computational techniques can aid in understanding the intramolecular interactions that occur in the selected compounds. Methods such as the molecular electrostatic potential (MEP)^14^ and the noncovalent interaction (NCI) index^51^ are powerful tools for visualizing regions prone to interactions and highlighting weak contacts in molecular systems. However, in the present study, our main objective was to establish a direct correlation between specific molecular interactions, particularly intramolecular pnictogen bonds, and the NMR parameters of interest. To this end, we employed AIM, NBO, and EDA analyses, as these techniques offer a more detailed and quantitative assessment of molecular interactions. These methods enable a comprehensive evaluation of interaction topology, orbital contributions, and energy decomposition, providing insights that go beyond those afforded by MEP and NCI analyses. Accordingly, AIM, NBO, and EDA are particularly well-suited for elucidating the electronic nature of the interactions under investigation as well as their influence on the associated NMR parameters.

The topological parameters obtained from the atom-in-molecule (AIM) analysis at the bond critical points (BCP) offer valuable insights into the existence, nature, and strength of the interaction. Figure 2 brings information about the location of the BCP related to the intramolecular interaction with the four select compounds.

**Figure 2:**
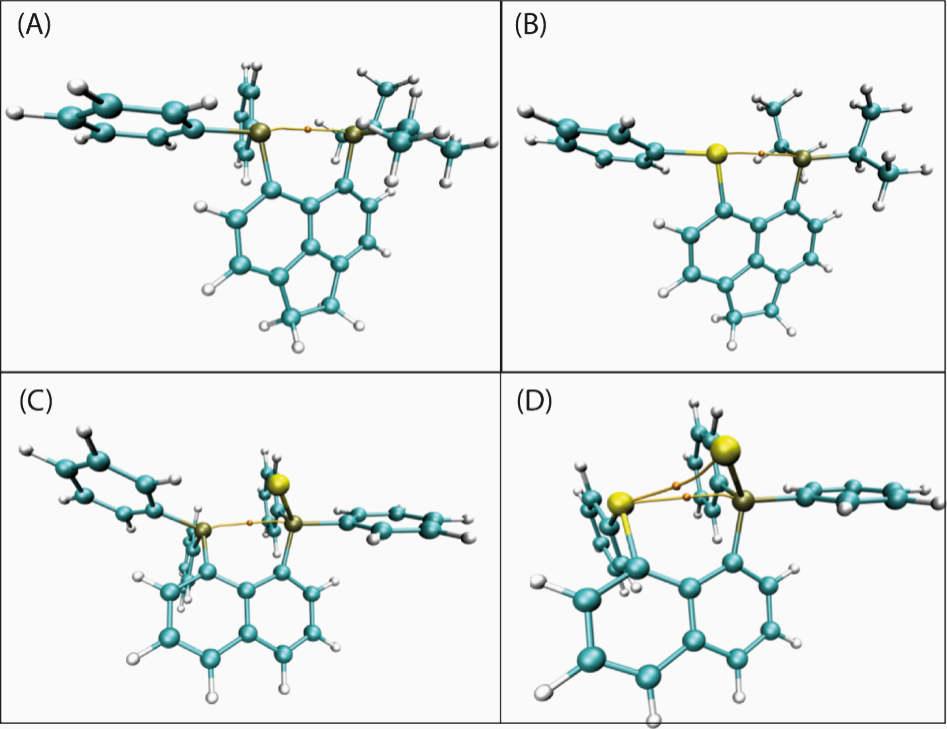
Bond critical points related to the intramolecular interactions for compounds **1** (A), **2** (B), **3** (C), and **4** (D).

The presence of a BCP between the P—P, compounds **1** and **3**, or P—Se, compounds **2** and **4**, is axis evidence of the existence of an intramolecular interaction between these atoms. For compounds **1** and **3**, this interaction may be characterized as a pnictogen bond since the interaction occurs between two pnictogen atoms. On the other hand, for compounds **2** and **4**, both pnictogen and chalcogen bonds can occur. Also, in Figure 2, part D, it is possible to observe a BCP in the axis of Se—Se, pointing out an interaction between these atoms, i.e., a possible chalcogen bond. Then, it is necessary to understand the nature of this interaction.

Table 2 presents the values for the AIM parameters for the selected compounds. It is possible to compare the interaction of compounds **1** and **3**, with compounds **2** and **4**, respectively. The values indicate that for both pairs of compounds, *i.e*., **1** and **2**, and **3** and **4**, the intramolecular contact has similar characteristics. Similar values of ρ and ∇^2^ρ are observed for both pairs at the BCP that correspond to PnB, indicating that both compounds present a similar strength.

**Table 2:** Topological Parameters (a.u.) at the Bond Critical Point (BCP) that Correspond to the Intramolecular Interactions^a^

compound	ρ	∇^2^ρ	*V*	*G*	*–G*/*V*	*E*_int_
**1**	0.0216	0.0358	–0.0109	0.0099	0.9095	–0.0055
**2**	0.0244	0.0447	–0.0134	0.0123	0.9169	–0.0067
**3**	0.0164	0.0372	–0.0089	0.0091	1.0203	–0.0045
**4**	0.0173	0.0387	–0.0095	0.0096	1.0073	–0.0048

a ρ = electron density; ∇^2^ρ = Laplacian of the electron density; *V* = potential energy; *G* = kinetic energy.

To better understand the nature of these interactions, natural bond orbital (NBO) analysis can provide insights into the orbitals involved in the interaction and the associated energy. In Figure 3, it is possible to observe the donating orbitals, corresponding to the lone pair of P or Se, and the receiving orbitals, i.e., the σ*­(P—C). Table S1 brings the main orbital contributions.

**Figure 3:**
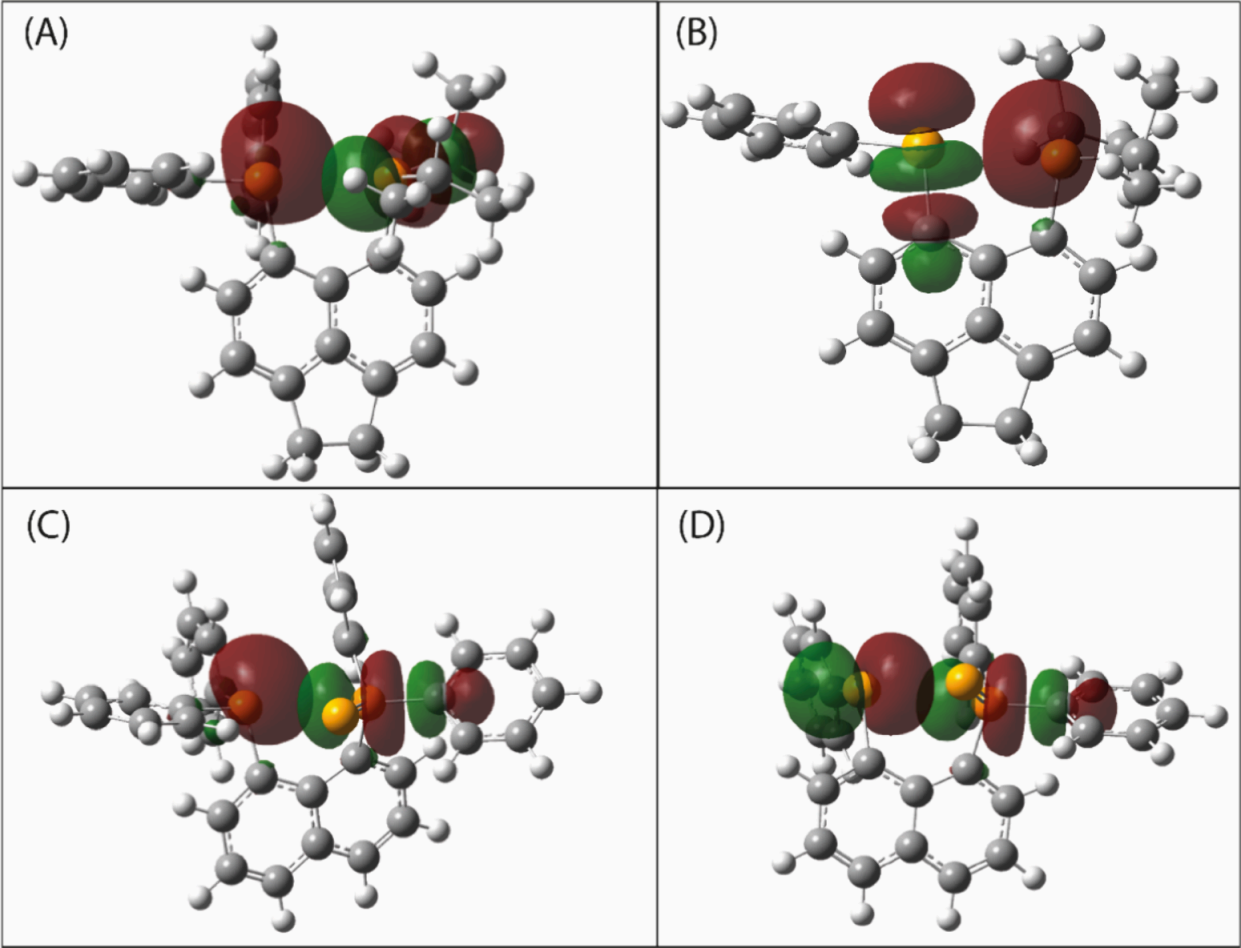
NBOs involved in the interaction of P—P, for compounds **1** (A) and **3** (C), or P—Se, for compounds **2** (B) and **4** (D).

The most stabilizing donor–acceptor interaction is the one that is responsible for the intramolecular interaction and, where applicable, the definition of the presence of a pnictogen or chalcogen bond. For compound **1**, the energy related to the interaction is around 2.56 kcal/mol. For compound **2**, the most significant interaction presents an energy of 7.76 kcal/mol between the P lone pair and σ*­(Se—C), indicating the presence of a chalcogen bond. Compounds **3** and **4**, like compound **1**, exhibit a PnB interaction with slightly higher stabilization energies of 3.94 and 4.23 kcal/mol, respectively. These values suggest that the electronic features of these systems are very similar in nature.

Recent findings point out that intramolecular interactions, like the pnictogen bond and chalcogen bond, show an important covalent factor in the interaction energy.^16,17,20^ In this sense, performing an energy decomposition analysis (EDA) is essential to understanding which contributions are most relevant.

The decomposition used in this work splits the interaction energy in three terms (eq S1), the Pauli repulsion energy, the Coulomb interaction, i.e., the electrostatic interaction, and the orbital interaction energy (*E*_OI_). Our EDA calculations point out that *E*_OI_ is the most important factor in the total interaction of energy. For compounds **1**, **3**, and **4**, this factor represents 72.10, 70.47, and 70.43%, respectively, and for compound **2**, this percentage corresponds to 71.99%. This additional perspective confirms the similarity between PnB that occurs in compounds **1**, **3**, and **4**. These results are in agreement with values reported in the literature, where the orbital contribution to the interaction energy for the pnictogen bond ranges from around 62 to 82% for organophosphorus compounds^20^ and 33 to 65% for model structures,^17^ and 37 to 72% for model structures that present the chalcogen bond.^18^ These findings reinforce the thesis that these interactions are not electrostatic but rather covalent.

In short, the AIM, NBO, and EDA results indicate that the nature of the pnictogen bond is similar for compounds **3** and **4**. Also, compound **1** presents an intramolecular PnB, while compound **2** displays a chalcogen bond. Therefore, these parameters are not the main values that govern the NMR parameters in the compounds studied. Considering the potential of these molecules as qubit candidates, they should exhibit a high SSCC value and a significant difference between the δ values of the nuclei involved.^1^

Our goal in this work is to study the influence of the pnictogen bond in the NMR parameters. In this context, compounds **1** and **3** were selected for the next stage of the study, aiming to better understand the role of the interactions between phosphorus atoms—specifically the pnictogen bond—in influencing the NMR parameters and to enable comparison of this effect across similar molecular structures.

### Influence of the Pnictogen Bond in the NMR Parameters

As mentioned earlier, compounds **1** and **3** were selected for this step of the work. In addition to the facts already described, another factor that influenced the choice of these compounds was the guarantee that the interaction between the nuclei involved in the NMR parameters will be a pnictogen bond, since we have two phosphorus atoms, so surely one will act as the Lewis acid and the other as the Lewis basis.

The distance between the phosphorus atoms was changed from the crystallographic structure and increased until the AIM analysis showed that there is no more BCP between the phosphorus; i.e., the pnictogen bond no longer exists and decreased until around 2.20 Å, that is, the P—P bond distance. For compound **1**, the original distance between phosphorus is 3.09 Å, and they have been moved away from each other to 4.39 Å and closer together to 2.19Å. These distances for compound **3** have a smaller range, going from 2.20 to 3.40 Å, with the crystallographic structure showing the distance between P of 3.20 Å.

The chemical shift values can be obtained in Tables S2 and S3. For compound **1**, it is possible to observe that the chemical shifts for P1 are higher when the phosphorus is closer to each other and closer to zero when the distance is the maximum. For P2, the behavior is the opposite, with δ showing the lowest values with the phosphorus closer to each other, and the maximum value with the distance between the P equal to 4.39 Å. In this scenario, the interaction energy and the electron density have the highest values with the lower P—P distance (Figure 5A), so the phosphorus that acts as a donor, P1, has a negative Δδ along the distance, and for the Lewis base, P2, the Δδ is positive, with negative δ values.

For compound **3**, the behavior is quite similar, with the values of δ for P1 decreasing when the atoms come closer and increasing for P2. However, the values of δ for compound **3** are always positive. As seen in compound **1**, Δδ­(P1) is negative; however, the variation of the chemical shift of P2, i.e., the phosphorus that receives electron density, is also negative. An interesting behavior can be observed for the two phosphorus atoms in both compounds, where when these atoms are brought closer together, thus increasing the interaction energy and the electron density (Figure 5), the shift values move away from zero, becoming larger for both P in compound **3** and P1 in compound **1**, and decreasing for P2 in compound **1.**

This behavior between donor and acceptor atoms can be interpreted through Ramsey’s theory of nuclear shielding, in which the chemical shift arises from the sum of diamagnetic and paramagnetic contributions to the shielding tensor.^52,53^ The paramagnetic term, in particular, is sensitive to changes in orbital occupancy and electron delocalization caused by intramolecular interactions such as pnictogen bonding.^52,53^ As the interaction strengthens and the atoms move closer, the electronic coupling between donor and acceptor orbitals alters its energy, enhancing or suppressing paramagnetic deshielding, depending on the electronic role of each phosphorus atom. Therefore, the observed trends in Δδ reflect not only the proximity or electron density variations but also orbital effects that modulate shielding via the paramagnetic term of Ramsey’s framework.

In Figure 4, the relation between the distance and the spin–spin coupling constant can be observed. Part A is related to compound **1**, while part B shows the results for compound **3**. The shape of the curve for these compounds is very similar with higher values when the phosphorus atoms are closer. For compound **3**, the higher values of *J* are around 500 Hz, almost three times smaller than the higher values of compound **1**. Therefore, for both compounds, the increase in the strength of PnB causes an increase in the values of the SSCC.

**Figure 4:**
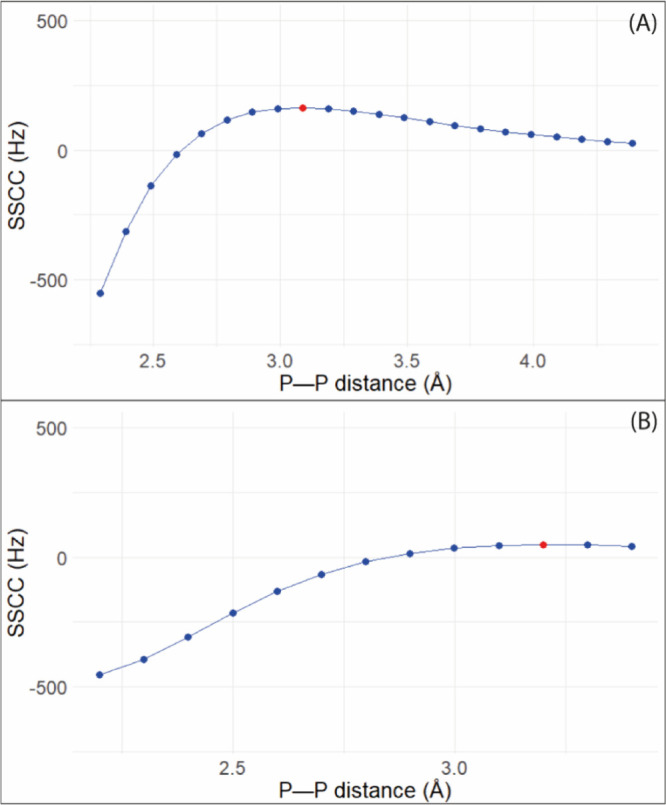
SSCC for compounds **1** (A) and **3** (B). The red point is the value for the original P—P distance.

This effect can be primarily attributed to changes in the Fermi contact (FC) term, which is the dominant contribution to the spin–spin coupling constants.^52,54,55^ As the pnictogen bond becomes stronger and the atoms become closer, the spin density at the phosphorus nuclei increases. This modulation of the electronic structure leads to an increase in the FC term and, consequently, in the observed *J* values. The sign inversion of the SSCC as the P—P distance changes is an intriguing characteristic seen in both compounds. Longer distances result in a positive coupling, while smaller distances yield negative values. This behavior may result from the competition between different Ramsey contributions to the total *J* coupling. At shorter distances, the spin–dipole or paramagnetic spin–orbit terms may contribute more significantly and negatively, while at longer distances, the FC term becomes dominant and positive. The SSCC’s sensitivity to structural changes is further supported by the change in the balance between contributions, which reflects alterations in the electronic environment and orbital interactions as a function of distance.

To help in understanding the behavior of the NMR parameters for both compounds, the nature of the P—P interaction was analyzed through its topological parameters and the decomposition of the energy related to the interaction. [Fig fig5] brings this information for compounds **1** and **3**, the topological parameters, ρ and *E*_int_, related to the P—P distance; the interaction energy (*E*_int_) values were obtained from the AIM parameters, specifically as half the potential energy value ( $E_{\mathrm{int}}=\frac{1}{2}V$ ), as described by Espinosa et al.^56^ This definition, initially established for hydrogen bonds, can be extended to other intermolecular interactions, such as the pnictogen bond, due to their similar nature. Values of ∇^2^ρ and *V* for the BCP of each P—P distance can be found in Tables S3 and S4. A similar behavior can be observed for both compounds, where the interaction energy is higher when the distance between the P comes closer to each other; the same occurs with the electronic density related to the BCP corresponding to the PnB.

**Figure 5:**
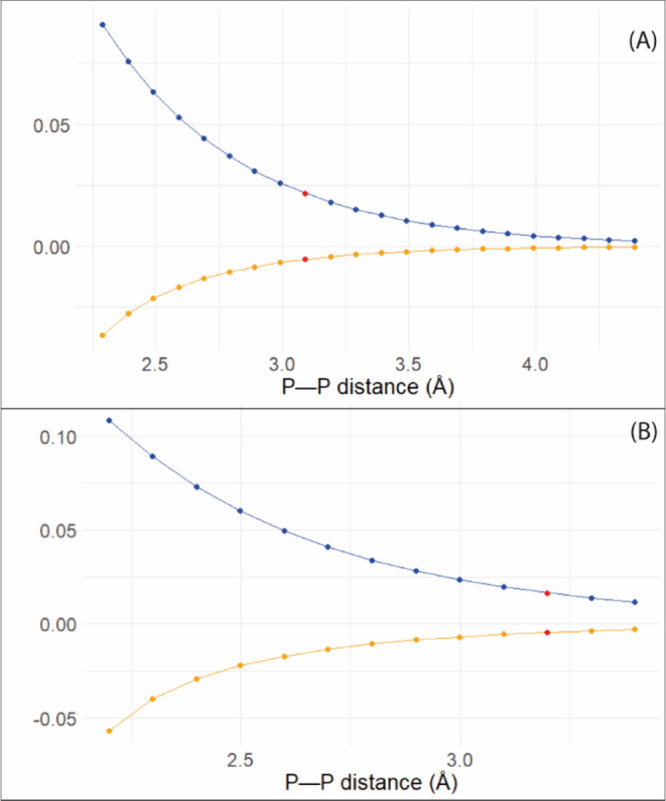
Topological parameters, ρ (blue, a.u.) and *E*_int_ (orange, kcal/mol), for compounds **1** (A) and **3** (B). The red point is the value for the original P—P distance.

The AIM analysis shows that, for compound **3**, the interaction between the phosphorus ends with just 0.2 Å further away from the crystallographic structure; for compound **1**, this distance was increased by 1.3 Å. When these distances were increased by another 0.1 Å, the AIM analysis no longer showed a BCP between the P atoms, indicating that the pnictogen bond no longer existed. For the scenario where the phosphorus atoms came closer to each other, for compound **1**, the distance of the P atoms decreased by 0.9 and 1.0 Å for compound **3**, reaching around 2.2 Å, the distance where a P—P bond is observed, so the intramolecular interaction does not exist.

Figure 6 provides information about the interaction energy, specifically the portion of this energy that came from the interaction between the phosphorus orbitals, provided by energy decomposition analysis (EDA). It is possible to observe that, as expected, the orbital interaction became stronger as the P—P distance became smaller. As seen for the crystallographic structure of compounds **1**–**4**, the contribution of the orbital interaction to the total interaction energy is the main factor and is primarily responsible for the interaction between the phosphorus atoms. The percentage of the orbital energy in the total interaction energy stays around 70% for compounds **1** and **3**. These values are in line with reported values for other pnictogen bonds.^17,20^

**Figure 6:**
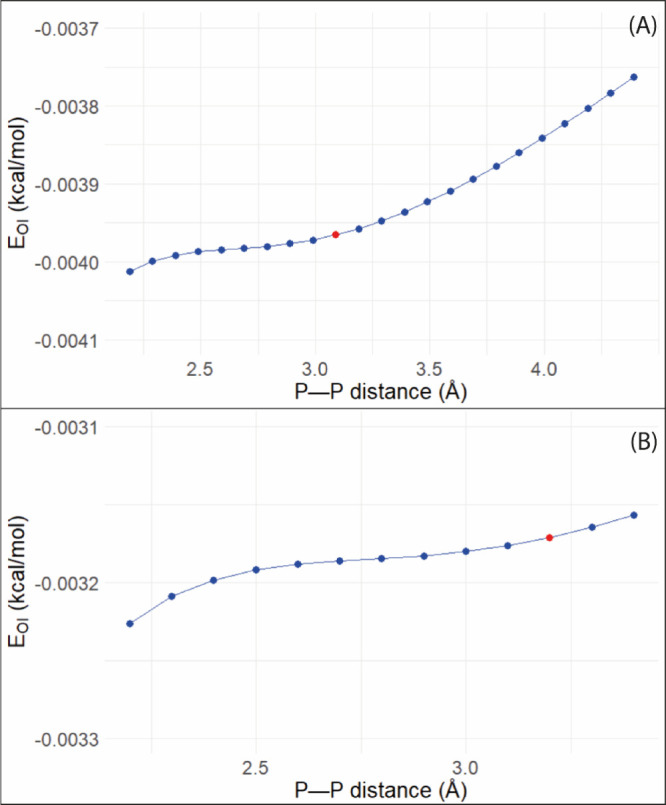
Orbital interaction energy for compounds **1** (A) and **3** (B). The red point is the value for the original P—P distance.

Thus, these results indicate that the interaction became stronger when the phosphorus atoms were closer to each other. Aligned with this, the difference between the δ for the two phosphorus atoms increases and the *J*_PP_ reaches the highest value when the interaction energy reaches the highest values. As a result, as the interaction became stronger, the NMR parameters reached values that make these compounds more promising for use as a QIP molecule.

These results reinforce the fact that the strength and nature of the intramolecular interaction are directly related to the NMR parameters of interest for qubit applications. As the interaction energy increases, the spin–spin coupling constants become larger and the chemical shifts are more differentiated, which improves the ability of the molecule to act as a qubit. Therefore, modulating these interactions through molecular design may serve as an effective strategy to enhance the NMR properties of these systems for quantum information processing (QIP).

## Conclusions

The importance of NMR parameters for a molecule to act as a quantum bit (qubit) in quantum information processing (QIP) is evident, highlighting the necessity for these molecules to meet specific criteria. The results in this work show that the four selected molecules meet those criteria. The AIM analysis points out the existence of intramolecular interactions occurring between the phosphorus atoms, for compounds **1** and **3**, and phosphorus and selenium for compounds **2** and **4**, and the NBO analysis indicates that these interactions can be characterized as a pnictogen bond (PnB) for compounds **1**, **3**, and **4**, and a chalcogen bond in compound **2**. Allied with these findings, an energy decomposition analysis (EDA) of the interaction energy (*E*_int_) shows that the main component of *E*_int_ is the orbital interaction energy, with around 70% of the total energy contribution.

In the sequence, the relationship between the strength of PnB and the NMR parameters was studied for compounds **1** and **3**. This current study demonstrates that, as the interaction energy of PnB increases, the spin–spin coupling constant reaches higher values and the chemical shifts for the phosphorus involved in the interaction are more distant from each other. The EDA analysis confirms that orbital interaction is the main factor for the interaction energy during the variation of distance analyzed. These behaviors increase the ability of the molecule to act as a qubit; i.e., as the energy of the PnB interaction between the two atoms relevant to the NMR parameters for qubits increases, the molecule aligns more closely with the required NMR parameters, enhancing its potential as a qubit in QIP. Furthermore, the use of those methods was essential to access interaction energies, topological properties, and orbital contributions, allowing for a detailed characterization of the bonding nature and its direct influence on spectroscopic parameters. These findings reinforce the role of theoretical approaches in understanding and predicting how specific intramolecular interactions can be employed to tune properties relevant to quantum applications.

## Supplementary Material



## Notes

Energy decomposition equation, Cartesian coordinates, AIM parameters, NBO parameters, and calculated chemical shifts (PDF)
