# Supplementary material for: Intramolecular Pnictogen Bonds as Key Determinants for NMR Quantum Computation Parameters
Source: ACS Omega. 2025 Sep 16;10(38):44201–9. doi: 10.1021/acsomega.5c05640 (PMC12489845; doi:10.1021/acsomega.5c05640)
Supplement: Supplementary file 1 [file ao5c05640_si_001.pdf]

Intramolecular Pnictogen Bonds as Key Determinants for NMR Quantum Computation Parameters

Gustavo A. Andolpho[1] *and Teodorico C. Ramalho*[1,2]*

[1]Chemistry Department, Institute of Natural Sciences, Lavras Federal University, 37200-900, Lavras, MG, Brazil.

[2]Center for Basic and Applied Research, University Hradec Kralove, Hradec Kralove, Czech Republic.

Equation S1. Decomposition of the Interaction Energy.

$$\Delta E_{int}(\xi) = \Delta V_{elestat}(\xi) + \Delta E_{Pauli}(\xi) + \Delta E_{OI}(\xi)$$

Table S1. Main results of the NBO analysis of the four compounds. Energy in kcal/mol. LP=Lone Pair.

Compound	Donor-Acceptor	Energy
1	LP(P1) — σ*(P2—C)	5.97
	LP(P2) — σ*(P1—C)	2.56
2	LP(Se) — σ*(P—C)	2.90
	LP(Se) — σ*(P—C)	1.75
	LP(Se) — σ*(P—C)	0.58
	LP(P) — σ*(Se—C)	7.76
3	LP(P1) — σ*(P2—C)	4.23
	LP(P1) — σ*(P2—C)	0.56
4	LP(Se) — σ*(P—C)	0.88
	LP(Se) — σ*(P—C)	3.94
	LP(Se) — σ*(P—C)	0.54

Table S2. Chemical Shifts values for compound **1**. Distance in Angstroms and chemical shifts in ppm.

Distance	δ(P1)	δ(P2)
4.39	0.77	-15.74
4.29	1.41	-15.74
4.19	1.81	-16.79
4.09	2.22	-17.87
3.99	2.39	-19.24
3.89	2.23	-20.29
3.79	1.78	-21.27
3.69	1.59	-21.68
3.59	1.76	-22.58
3.49	2.03	-24.11
3.39	2.37	-25.16
3.29	2.60	-25.65
3.19	2.92	-25.04
3.09	2.61	-23.71
2.99	3.34	-23.87
2.89	3.39	-24.57
2.79	3.55	-28.42
2.69	3.57	-29.84
2.59	3.51	-29.48
2.49	3.53	-29.52
2.39	3.57	.30.31
2.29	3.70	-31.07
2.19	4.29	-31.76

Table S3. Chemical Shifts values for compound **3**. Distance in Angstroms and chemical shifts in ppm.

**Distance**	**δ(P1)**	**δ(P2)**
3.40	70.29	2.72
3.30	72.51	2.78
3.20	72.99	1.57
3.10		
3.00	70.81	3.28
2.90	73.80	3.29
2.80	76.09	3.25
2.70	73.47	3.30
2.60	90.06	3.39
2.50	95.00	3.54
2.40	103.04	3.76
2.30	112.64	3.98
2.20	125.06	4.14

Table S4. Topological parameters for compound **1**. Distance in Angstroms and other parameters in atomic units. $\nabla^2\rho$=Laplacian of the electron density; V=Potential energy.

**Distance**	$\nabla^2\rho(r)$	**V**
4.39	0.0058	-0.0007
4.29	0.0066	-0.0008
4.19	0.0077	-0.0010
4.09	0.0089	-0.0012
3.99	0.0105	-0.0015
3.89	0.0122	-0.0019
3.79	0.0143	-0.0023
3.69	0.0166	-0.0029
3.59	0.0192	-0.0036
3.49	0.0221	-0.0045
3.39	0.0252	-0.0056
3.29	0.0287	-0.0070
3.19	0.0320	-0.0088
3.09	0.0358	-0.0109
2.99	0.0392	-0.0136
2.89	0.0418	-0.0170
2.79	0.0431	-0.0213
2.69	0.0419	-0.0267
2.59	0.0364	-0.0337
2.49	0.0240	-0.0430
2.39	0.0011	-0.0555
2.29	-0.0372	-0.0728
2.19	-0.0974	-0.0978

Table S5. Topological parameters for system **3**. Distance in Angstroms and other parameters in atomic units. $\nabla^2\rho$=Laplacian of the electron density; V=Potential energy.

**Distance**	**$\nabla^2\rho(r)$**	**V**
3.40	0.0287	-0.0059
3.30	0.0328	-0.0072
3.20	0.0372	-0.0089
3.10	0.0416	-0.0110
3.00	0.0459	-0.0137
2.90	0.0494	-0.0170
2.80	0.0514	-0.0213
2.70	0.0503	-0.0268
2.60	0.0442	-0.0342
2.50	0.0298	-0.0443
2.40	0.0025	-0.0586
2.30	-0.0440	-0.0796
2.20	-0.1168	-0.1134

Table S6. Cartesian coordinates (in Å) for compounds **1-4.**

Compound **1**			
P	-0.385	7.646	8.023
P	-2.834	8.214	6.223
C	-1.097	8.994	9.037
C	-0.533	9.295	10.264
C	-0.921	10.376	11.072
C	-1.900	11.207	10.603
C	-2.501	10.918	9.366
C	-3.474	11.884	9.051
C	-4.173	11.749	7.888
C	-3.921	10.618	7.088
C	-2.981	9.645	7.376
C	-2.174	9.806	8.555
C	-2.510	12.447	11.190
C	-3.534	12.922	10.134
C	0.285	6.448	9.271
C	1.057	5.366	8.525
C	-0.865	5.847	10.066
C	1.144	8.469	7.336
C	2.194	8.917	8.337
C	0.725	9.625	6.436
C	-4.279	8.487	5.116
C	-4.009	9.085	3.886
C	-5.026	9.330	2.974
C	-6.329	8.958	3.274
C	-6.607	8.345	4.488
C	-5.591	8.114	5.404
C	-3.486	6.841	7.241
C	-3.272	5.546	6.773
C	-3.765	4.452	7.467
C	-4.472	4.640	8.648
C	-4.682	5.925	9.126
C	-4.193	7.019	8.425
H	0.272	8.671	10.632
H	-0.431	10.544	12.024
H	-4.929	12.463	7.581
H	-4.526	10.504	6.199
H	-1.748	13.205	11.391
H	-2.990	12.228	12.147
H	-4.542	13.001	10.549
H	-3.279	13.910	9.742
H	0.974	6.946	9.960

H	0.416	4.861	7.797
H	1.424	4.613	9.229
H	1.920	5.770	7.991
H	-0.489	5.092	10.763
H	-1.588	5.364	9.403
H	-1.397	6.605	10.644
H	1.578	7.687	6.701
H	1.805	9.699	8.994
H	3.057	9.334	7.806
H	2.556	8.097	8.960
H	1.596	10.031	5.912
H	0.284	10.434	7.025
H	-0.011	9.314	5.692
H	-2.988	9.364	3.643
H	-4.799	9.802	2.024
H	-7.125	9.139	2.560
H	-7.623	8.048	4.725
H	-5.823	7.642	6.351
H	-2.706	5.395	5.859
H	-3.591	3.450	7.090
H	-4.852	3.785	9.196
H	-5.231	6.078	10.049
H	-4.366	8.020	8.805
Compound **2**			
Se	0.151	5.863	2.040
P	1.637	3.233	1.862
C	1.400	6.301	0.634
C	1.266	7.530	0.022
H	0.452	8.177	0.322
C	2.145	8.005	-0.967
H	1.981	8.987	-1.397
C	3.185	7.207	-1.351
C	3.313	5.940	-0.757
C	4.418	5.250	-1.286
C	4.689	3.991	-0.831
H	5.527	3.413	-1.203
C	3.836	3.438	0.142
H	4.058	2.432	0.473
C	2.739	4.088	0.677
C	2.448	5.417	0.231
C	4.288	7.417	-2.349
H	4.906	8.276	-2.075
H	3.888	7.625	-3.345
C	5.100	6.101	-2.320
H	5.100	5.605	-3.294
H	6.148	6.274	-2.059

C	-0.349	7.659	2.538
C	-1.647	8.091	2.302
H	-2.345	7.446	1.779
C	-2.047	9.352	2.728
H	-3.062	9.686	2.541
C	-1.149	10.183	3.380
H	-1.459	11.168	3.708
C	0.151	9.749	3.615
H	0.854	10.394	4.130
C	0.549	8.487	3.204
H	1.560	8.144	3.395
C	2.531	3.589	3.466
H	2.744	4.659	3.358
C	1.606	3.429	4.665
H	2.097	3.799	5.570
H	1.342	2.383	4.841
H	0.680	3.992	4.527
C	3.848	2.857	3.655
H	3.695	1.782	3.781
H	4.354	3.223	4.554
H	4.522	3.007	2.809
C	1.919	1.433	1.507
H	2.985	1.191	1.560
C	1.191	0.591	2.549
H	1.249	-0.466	2.273
H	0.133	0.863	2.607
H	1.623	0.698	3.544
C	1.396	1.115	0.113
H	0.329	1.345	0.040
H	1.523	0.050	-0.102
H	1.914	1.681	-0.663
Compound **3**			
Se	3.037	1.402	1.415
Se	5.059	3.617	2.914
P	4.558	1.999	4.201
C	2.805	1.995	4.720
C	2.606	1.893	6.080
C	1.361	2.128	6.680
C	0.321	2.547	5.908
C	0.458	2.644	4.506
C	-0.652	3.065	3.744
C	-0.589	3.118	2.383
C	0.575	2.676	1.740
C	1.672	2.250	2.448
C	1.690	2.292	3.870
C	5.490	2.205	5.752

C	6.130	1.134	6.368
C	6.837	1.331	7.547
C	6.900	2.590	8.121
C	6.256	3.661	7.511
C	5.560	3.471	6.332
C	5.024	0.337	3.661
C	4.283	-0.767	4.068
C	4.691	-2.042	3.706
C	5.837	-2.216	2.944
C	6.579	-1.111	2.544
C	6.176	0.164	2.903
C	2.267	-0.349	1.337
C	1.240	-0.783	2.164
C	0.821	-2.106	2.108
C	1.424	-3.000	1.235
C	2.449	-2.561	0.406
C	2.870	-1.242	0.454
H	3.440	1.659	6.727
H	1.256	2.024	7.753
H	-0.636	2.803	6.349
H	-1.560	3.338	4.271
H	-1.438	3.451	1.797
H	0.600	2.630	0.658
H	6.083	0.144	5.933
H	7.340	0.492	8.015
H	7.452	2.741	9.042
H	6.303	4.649	7.956
H	5.071	4.309	5.847
H	3.384	-0.634	4.657
H	4.105	-2.900	4.014
H	6.150	-3.213	2.657
H	7.474	-1.244	1.947
H	6.743	1.032	2.583
H	0.765	-0.101	2.858
H	0.019	-2.437	2.758
H	1.095	-4.032	1.197
H	2.925	-3.249	0.283
H	3.675	-0.909	0.193
Compound **4**			
Se	6.748	1.029	2.650
P	6.116	2.770	3.690
P	3.445	2.012	2.101
C	5.729	4.205	2.620
C	6.311	5.389	3.019
H	6.769	5.461	3.996
C	6.395	6.507	2.175

H	6.850	7.419	2.541
C	5.981	6.394	0.882
H	6.118	7.209	0.179
C	4.929	5.131	-0.914
H	5.152	5.961	-1.576
C	4.254	4.034	-1.366
H	3.946	3.959	-2.402
C	3.879	3.040	-0.451
H	3.227	2.241	-0.786
C	4.256	3.087	0.871
C	5.111	4.131	1.328
C	5.348	5.216	0.431
C	7.536	3.338	4.681
C	7.426	3.612	6.039
H	6.474	3.509	6.544
C	8.542	4.019	6.760
H	8.447	4.223	7.821
C	9.765	4.164	6.128
H	10.634	4.484	6.692
C	9.878	3.896	4.768
H	10.833	4.006	4.268
C	8.772	3.479	4.050
H	8.864	3.253	2.994
C	4.811	2.563	4.925
C	4.691	1.350	5.592
H	5.317	0.514	5.299
C	3.762	1.213	6.610
H	3.665	0.264	7.126
C	2.956	2.287	6.968
H	2.227	2.176	7.762
C	3.081	3.498	6.304
H	2.448	4.336	6.574
C	4.009	3.641	5.284
H	4.097	4.587	4.762
C	1.936	2.980	2.452
C	1.138	2.525	3.503
H	1.424	1.627	4.042
C	-0.009	3.209	3.870
H	-0.621	2.837	4.684
C	-0.361	4.379	3.209
H	-1.252	4.923	3.503
C	0.435	4.851	2.177
H	0.168	5.765	1.658
C	1.574	4.154	1.796
H	2.179	4.530	0.981
C	2.817	0.647	1.067

C	1.470	0.470	0.758
H	0.736	1.187	1.105
C	1.058	-0.618	0.000
H	0.006	-0.741	-0.233
C	1.986	-1.539	-0.465
H	1.663	-2.385	-1.061
C	3.331	-1.369	-0.163
H	4.064	-2.084	-0.521
C	3.742	-0.291	0.603
H	4.793	-0.165	0.845
